# Associations of multimorbidity burden with healthcare utilization and expenditures among individuals with diabetes in China

**DOI:** 10.3389/fpubh.2026.1975609

**Published:** 2026-10-28

**Authors:** Jielong Wu, Bo Li, Xuemin Wu, Xin Hu, Hengyuan Zhang, Yiling Li, Zhihai Zhang, Miaona Cai, Chunli Piao, Nengjiang Zhao, Shuyu Yang

**Affiliations:** 1 Center of Integrated Chinese and Western Medicine, The First Affiliated Hospital of Xiamen University, School of Medicine, Xiamen University, Xiamen, Fujian, China; 2 Department of Endocrinology, Shenzhen Hospital (Futian) of Guangzhou University of Chinese Medicine, Shenzhen, Guangdong, China; 3 Department of Nephrology, The First Affiliated Hospital of Xiamen University, School of Medicine, Xiamen University, Xiamen, Fujian, China

**Keywords:** Chinese population, diabetes, healthcare utilization, medical expenditures, multimorbidity

## Abstract

**Objective:**

Multimorbidity is common among people with diabetes, but its associations with healthcare utilization and medical expenditures in China remain insufficiently characterized. This study aimed to examine these associations using different measures of multimorbidity burden and assess potential sex differences.

**Methods:**

We conducted cross-sectional analyses using the 2018 China Health and Retirement Longitudinal Study (CHARLS) and a retrospective hospital-based cohort. Multimorbidity burden was assessed using four measures: multimorbidity, ≥3 LTCs, number of long-term conditions (LTCs), and the Chinese Multimorbidity-Weighted Index (CMWI). Multivariable regression models were used to examine associations with healthcare utilization and expenditures. Sex-stratified analyses, interaction tests, and sensitivity analyses were also performed.

**Results:**

Among 2,531 CHARLS participants with diabetes, 92.9% had multimorbidity and 77.7% had ≥3 LTCs. All four measures were positively associated with inpatient and outpatient utilization, visit counts, and medical expenditures. The presence of ≥3 LTCs was associated with higher odds of inpatient (OR, 2.44; 95% CI, 1.90–3.16) and outpatient utilization (OR, 2.17; 95% CI, 1.68–2.84), as well as higher total inpatient (cost ratio, 2.98; 95% CI, 1.94–5.24) and outpatient expenditures (cost ratio, 3.15; 95% CI, 1.92–5.72). In the hospital cohort (*n* = 3,963), all four measures were positively associated with hospitalization frequency, cumulative inpatient days, and inpatient expenditure. Most sex interaction tests were not statistically significant. Sensitivity analyses yielded generally consistent findings.

**Conclusion:**

Greater multimorbidity burden was associated with higher healthcare utilization and medical expenditures among individuals with diabetes across two different settings. These findings highlight the importance of considering coexisting conditions in healthcare planning.

## Introduction

Diabetes is a major global public health challenge, and China has the world’s largest population of people with diabetes (1). Population ageing, lifestyle changes, and increased life expectancy among people with diabetes have contributed to a growing burden of both traditional microvascular and macrovascular complications and other long-term conditions (LTCs), including cancer and cognitive impairment (2). Approximately one-third of people with diabetes develop at least three chronic conditions by 50 years of age (3–5).

Multimorbidity, commonly defined as the coexistence of two or more chronic conditions, complicates clinical care and is associated with functional decline and higher mortality (6–8). Among people with diabetes, cohort studies from the United Kingdom and Taiwan have linked a greater number of coexisting conditions to higher all-cause mortality (9). A US cohort study also found that increasing multimorbidity burden was associated with hypoglycemia-related emergency department visits and hospitalizations, including in analyses restricted to diabetes (10). People with multimorbidity also incur greater out-of-pocket expenditure on medicines than those without multimorbidity (11). Despite these clinical and financial consequences, the associations of specific comorbidity patterns with healthcare utilization and medical expenditures among people with diabetes remain insufficiently characterized in China.

Existing comorbidity indices, including the Charlson Comorbidity Index, vary in the conditions and severity categories they capture and may require clinical information that is not consistently available in population-based surveys (12, 13). The Chinese Multimorbidity-Weighted Index (CMWI) was developed using Chinese longitudinal data (14), yet its application in evaluating the economic fallout of diabetic comorbidities is limited. Beyond these methodological constraints, current multimorbidity research is further hindered by suboptimal data sources. Many studies rely heavily on either self-reported surveys—which are susceptible to recall bias—or localized administrative claims that lack national representativeness (15–17).

Understanding the associations between multimorbidity burden and healthcare utilization and expenditures among people with diabetes in China may help inform integrated care and healthcare resource planning. This study aimed to examine these associations across different measures of multimorbidity burden and assess whether they differed by sex. The findings may help identify the healthcare needs and expenditure burden associated with multimorbidity in this population.

## Materials and methods

### Study design and data sources

We combined a cross-sectional analysis of the 2018 China Health and Retirement Longitudinal Study (CHARLS) survey with supplementary analyses of a retrospective hospital cohort with one-year post-discharge follow-up (Figure 1).

**Figure 1:**
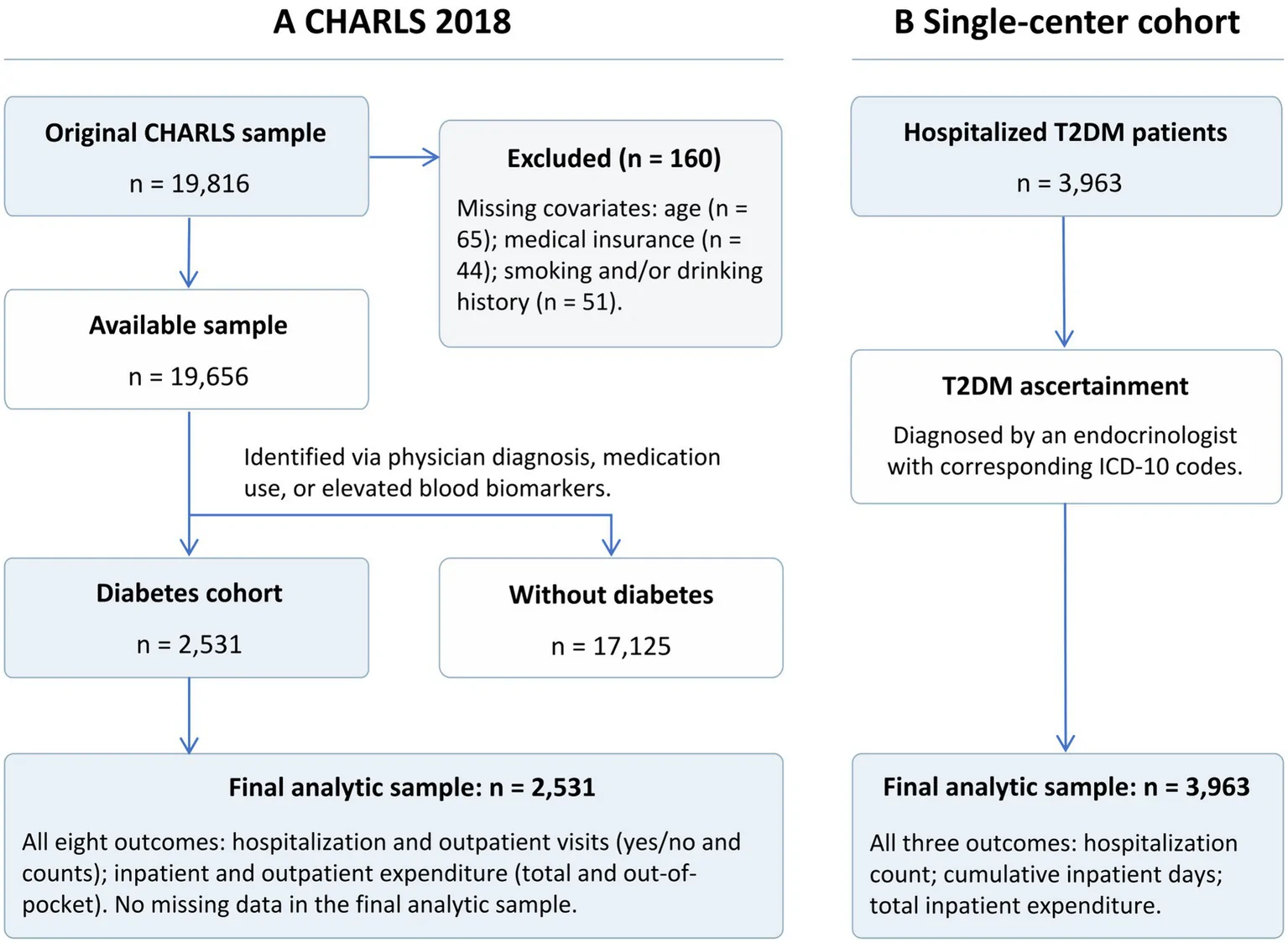
Participant selection in the CHARLS and hospital cohorts. CHARLS, China Health and Retirement Longitudinal Study.

Phase 1 utilized data from the 2018 wave of the CHARLS, a nationally representative longitudinal survey of middle-aged and older adults in China. Of the original 19,816 participants, 160 were excluded because of missing covariate data, leaving 19,656 participants, including 2,531 with diabetes. Data were collected via structured face-to-face interviews, covering comprehensive sociodemographic covariates, comorbidity profiles and healthcare burden. Diabetes in CHARLS was identified using self-reported physician-diagnosed diabetes or diabetes medication use recorded in 2018 or earlier survey waves, or fasting plasma glucose ≥7.0 mmol/L (126 mg/dL) or HbA1c ≥ 6.5% measured in the 2015 wave. Participants meeting any one of these criteria were classified as having diabetes. The comparator group comprised participants not classified as having diabetes under this algorithm and was labelled “participants without diabetes.” These criteria did not distinguish diabetes types.

Phase 2 used retrospective electronic medical records from Shenzhen Hospital (Futian), Guangzhou University of Chinese Medicine, a tertiary Grade-A hospital in Shenzhen. The cohort included 3,963 patients hospitalized between January 2018 and December 2023 who had type 2 diabetes mellitus (T2DM) diagnosed by endocrinologists with corresponding International Statistical Classification of Diseases and Related Health Problems, 10th Revision (ICD-10) E11 codes. All patients were followed for one year after their first discharge. Extracted data included sociodemographic characteristics, recorded discharge diagnoses, hospitalization count, cumulative inpatient days, and total inpatient expenditure. These analyses examined associations within hospitalized patients and did not constitute formal predictive validation.

The CHARLS study (Phase 1) was approved by the Ethical Review Committee of Peking University, and all participants provided written informed consent. For Phase 2, the study protocol was approved by the ethics committee of Shenzhen Hospital (Futian), Guangzhou University of Chinese Medicine. Given the retrospective nature of the study and the use of de-identified data, the requirement for informed consent was waived by the IRB.

### Measurement of comorbidity patterns

We focused on 14 common LTCs prevalent in the older Chinese population: hypertension, dyslipidaemia, diabetes, cancer, chronic lung diseases, liver disease, heart disease, stroke, kidney disease, digestive disease, psychiatric problems, memory-related diseases, arthritis, and asthma.

In CHARLS, non-diabetes LTCs were identified from self-reported physician diagnoses. Diabetes was ascertained using the criteria described above. In the hospital cohort, the corresponding conditions were identified from recorded discharge diagnoses using disease names and available ICD-10 codes. Supplementary Table S1 provides the conditions included within each hospital disease category and the corresponding ICD-10 codes. Each LTC category was coded as a binary indicator, with multiple diagnoses within the same category counted only once.

Based on the 14 selected LTCs, we constructed four distinct metrics to comprehensively evaluate multimorbidity burden: (1) the total Number of LTCs, (2) Multimorbidity status, (3) ≥ 3 LTCs, and (4) the CMWI. The Number of LTCs was calculated as a simple summation of diagnosed conditions for each individual. Multimorbidity was defined as the coexistence of at least two of these chronic conditions. We additionally assessed the presence of ≥3 LTCs, irrespective of the number of organ systems affected. The CMWI was calculated using disease-specific weights derived from Chinese longitudinal data (14). Besides, for descriptive comparisons between CHARLS participants with and without identified diabetes, non-diabetes LTC counts were calculated using the same 13 non-diabetes condition categories in both groups.

### Outcome measures

We assessed healthcare utilization and medical expenditures. In CHARLS, inpatient utilization and expenditure referred to the preceding year, whereas outpatient utilization and expenditure referred to the preceding month. Utilization outcomes included binary indicators of at least one inpatient admission or outpatient visit, together with hospitalization and outpatient visit counts during the corresponding recall periods. Expenditure outcomes comprised total and out-of-pocket expenditures for inpatient and outpatient services. In the hospital cohort, outcomes comprised hospitalization count, cumulative inpatient days, and cumulative total inpatient expenditure, including the index hospitalization and subsequent hospitalizations during the year following the first discharge. Only hospitalizations recorded at the study hospital were captured.

All expenditures were expressed in constant 2018 Chinese yuan (CNY). Hospital expenditures were adjusted using annual consumer price indices published by the National Bureau of Statistics of China, rebased to 2018. Each expenditure was multiplied by the ratio of the 2018 price index to the index for the year incurred before aggregation at the patient level. CHARLS expenditures from the 2018 survey were retained without further adjustment.

### Covariates

In Phase 1 (CHARLS), the primary models adjusted for age, sex, education level, residence, marital status, health insurance, and glycemic control status. Good self-reported glycemic control was defined as answering “Yes” to the question, “Is your blood sugar generally under control?” Smoking status, alcohol consumption, social activity participation, physical activity, and sleep duration were additionally included in sensitivity analyses because some of these variables may be consequences of multimorbidity rather than confounders. In Phase 2 (the hospital cohort), the primary models adjusted for age, sex, health insurance type, and recorded diabetes-related complications. Complications were identified from explicit discharge diagnosis names or ICD-10 codes E11.0-E11.8. E11.9, indicating T2DM without complications, was not classified as evidence of a complication. This variable captured the presence of recorded complications rather than their severity. Self-reported glycemic control and recorded diabetes-related complications were included to account partly for differences in diabetes-related clinical status; neither constituted a comprehensive measure of diabetes severity.

### Statistical analysis

First, participant characteristics, comorbidity prevalence, and healthcare utilization and expenditures were summarized as means ± standard deviations or counts and percentages. Comparisons between CHARLS participants with and without diabetes used Welch’s two-sample t tests for continuous variables and Pearson’s chi-square tests for categorical variables. Mean numbers of LTCs and mean numbers of non-diabetes LTCs were plotted across age groups by diabetes status to describe cross-sectional age-group differences.

Second, analyses were restricted to CHARLS participants with diabetes (*n* = 2,531) to examine associations between four multimorbidity measures and healthcare outcomes. Each measure was entered into a separate model, adjusted for the covariates described above. Logistic regression was used for binary inpatient and outpatient utilization outcomes, reporting odds ratios (ORs) and 95% confidence intervals (CIs). Hospitalization and outpatient visit counts were analyzed using quasi-Poisson regression with a log link, reporting count ratios and 95% CIs. The four expenditure outcomes were analyzed using two-part models, combining logistic regression for the probability of positive expenditure with Gamma regression using a log link among participants with positive expenditure. Zero expenditure values were retained in the first part. Predictions from both components were combined and standardized over the covariate distribution of the analyzed sample to estimate overall mean expenditure, including zero expenditure. Results were reported as overall cost ratios, with percentile-based 95% CIs obtained from 1,000 participant-level bootstrap resamples. Separate first-part ORs and second-part cost ratios were also reported.

Third, supplementary association analyses were conducted in the hospital cohort (*n* = 3,963). Hospitalization count was analyzed using zero-truncated negative binomial regression with a log link because all participants had at least one hospitalization. Exponentiated coefficients represented ratios of the underlying negative binomial mean parameter before truncation. Cumulative inpatient days and total inpatient expenditure were analyzed using Gamma regression with a log link, reporting adjusted mean ratios and 95% CIs. All patients had the same one-year post-discharge follow-up duration; therefore, no follow-up-time offset was included.

For both cohorts, binary multimorbidity measures were compared with their reference category; LTC count and CMWI associations were reported per additional condition and per 1-point increase, respectively. Sex-stratified analyses were conducted in both cohorts, omitting sex from the adjustment set. Formal interaction tests included terms between sex and each multimorbidity measure in the full-sample models. For two-part expenditure models, interaction terms were jointly tested across the two components. All sex-stratified analyses and interaction tests were exploratory. Interaction *p* values were nominal and unadjusted for multiple testing; findings were considered hypothesis-generating rather than confirmatory evidence of effect modification by sex.

Finally, sensitivity analyses assessed the robustness of the findings. Primary CHARLS analyses were unweighted and focused on associations within the analytic sample. Survey-weighted sensitivity analyses incorporated 2018 individual weights, community clustering, and county-level stratification as an approximate survey-design specification. Weighted cost CIs were calculated using bootstrap replicate-weight standard errors on the log-ratio scale. Further CHARLS analyses additionally adjusted for the five lifestyle variables and excluded diabetes from all four multimorbidity measures. Hospital sensitivity analyses excluded diabetes from these measures and additionally adjusted for pandemic period, categorized as 2018–2019 versus 2020–2023. When diabetes was excluded, multimorbidity and ≥3 LTCs were recalculated using thresholds of at least two and three non-diabetes LTCs, respectively, and the assigned diabetes weight was subtracted from CMWI. Sensitivity analyses examined overall associations without sex stratification or interaction testing.

Analyses were performed using R version 4.4.1. All tests were two-sided, with *p* values <0.05 considered statistically significant.

## Results

### Participant characteristics and multimorbidity burden

Table 1 summarizes the characteristics of the 19,656 CHARLS participants, including 2,531 (12.88%) with diabetes. Compared with participants without diabetes, those with diabetes were older (mean age, 63.49 vs. 61.13 years), more likely to be women (56.18% vs. 52.28%), and more likely to live in urban areas (49.47% vs. 38.93%; all *p* < 0.001). The groups also differed in education, health insurance, smoking, alcohol consumption, physical exercise, sleep duration, and participation in social activities. Among participants with diabetes, 67.44% reported good glycemic control.

**Table 1:** Characteristics of CHARLS participants by diabetes status.

Characteristic	Overall	No Diabetes	Diabetes	*p* value
	*N* = 19,656	*N* = 17,125	*N* = 2,531	
Demographics				
Sex (female)	10,375 (52.78%)	8,953 (52.28%)	1,422 (56.18%)	<0.001
Age (years)	61.43 ± 10.40	61.13 ± 10.49	63.49 ± 9.52	<0.001
Illiterate or primary education	17,157 (87.29%)	15,005 (87.62%)	2,152 (85.03%)	<0.001
Urban residence	7,918 (40.28%)	6,666 (38.93%)	1,252 (49.47%)	<0.001
Married	15,419 (78.44%)	13,430 (78.42%)	1,989 (78.59%)	0.853
Public health insurance	18,913 (96.22%)	16,446 (96.04%)	2,467 (97.47%)	<0.001
Current smoking	8,270 (42.07%)	7,266 (42.43%)	1,004 (39.67%)	0.009
Current drinking	9,312 (47.37%)	8,185 (47.80%)	1,127 (44.53%)	0.002
Participation in social activities	8,799 (44.76%)	7,602 (44.39%)	1,197 (47.29%)	0.006
Physical activity	11,899 (60.54%)	10,547 (61.59%)	1,352 (53.42%)	<0.001
Sleep duration (hours)				0.024
≤6	7,481 (38.06%)	6,461 (37.73%)	1,020 (40.30%)	
6–8	7,224 (36.75%)	6,348 (37.07%)	876 (34.61%)	
>8	4,951 (25.19%)	4,316 (25.20%)	635 (25.09%)	
Good glycemic control			1,707 (67.44%)	
Comorbidity				
Hypertension	7,538 (38.35%)	5,917 (34.55%)	1,621 (64.05%)	<0.001
Dyslipidaemia	4,331 (22.03%)	2,996 (17.49%)	1,335 (52.75%)	<0.001
Cancer or malignant tumor	448 (2.28%)	359 (2.10%)	89 (3.52%)	<0.001
Chronic lung diseases	3,043 (15.48%)	2,536 (14.81%)	507 (20.03%)	<0.001
Liver disease	1,367 (6.95%)	1,107 (6.46%)	260 (10.27%)	<0.001
Heart disease	3,882 (19.75%)	2,983 (17.42%)	899 (35.52%)	<0.001
Stroke	1,417 (7.21%)	1,056 (6.17%)	361 (14.26%)	<0.001
Kidney disease	1,959 (9.97%)	1,514 (8.84%)	445 (17.58%)	<0.001
Stomach or other digestive disease	5,928 (30.16%)	5,079 (29.66%)	849 (33.54%)	<0.001
Emotional, nervous, or psychiatric problems	644 (3.28%)	516 (3.01%)	128 (5.06%)	<0.001
Memory-related diseases	792 (4.03%)	592 (3.46%)	200 (7.90%)	<0.001
Arthritis or rheumatism	7,664 (38.99%)	6,525 (38.10%)	1,139 (45.00%)	<0.001
Asthma	1,162 (5.91%)	950 (5.55%)	212 (8.38%)	<0.001
Number of non-diabetes long-term conditions	2.04 ± 1.81	1.88 ± 1.71	3.18 ± 2.06	<0.001
Number of long-term conditions	2.17 ± 1.92	1.88 ± 1.71	4.18 ± 2.06	<0.001
Multimorbidity	11,008 (56.00%)	8,656 (50.55%)	2,352 (92.93%)	<0.001
≥3 long-term conditions	7,072 (35.98%)	5,106 (29.82%)	1,966 (77.68%)	<0.001
Chinese multimorbidity-weighted index	3.28 ± 3.32	2.90 ± 3.04	5.80 ± 3.95	<0.001
Healthcare utilization and expenditures				
Hospitalization in the past year	3,310 (16.84%)	2,623 (15.32%)	687 (27.14%)	<0.001
Outpatient visits in the past month	3,239 (16.48%)	2,657 (15.52%)	582 (22.99%)	<0.001
Number of hospitalizations in the past year	0.28 ± 1.37	0.25 ± 1.40	0.47 ± 1.07	<0.001
Number of outpatient visits in the past month	0.36 ± 1.36	0.33 ± 1.32	0.51 ± 1.57	<0.001
Total inpatient expenditure in the past year (CNY)	2,652.92 ± 17,407.09	2,284.17 ± 16,657.63	5,147.94 ± 21,651.22	<0.001
Out-of-pocket inpatient expenditure in the past year (CNY)	1,378.62 ± 10,595.48	1,211.27 ± 10,420.01	2,510.92 ± 11,653.12	<0.001
Total outpatient expenditure in the past month (CNY)	283.96 ± 3,269.55	265.54 ± 3,337.48	408.63 ± 2,763.83	0.018
Out-of-pocket outpatient expenditure in the past month (CNY)	177.59 ± 1,749.03	170.40 ± 1,817.14	226.23 ± 1,188.97	0.042

Data are presented as *n* (%) or mean ± SD. *p* values compare participants with and without diabetes and were calculated using Pearson’s chi-square test for categorical variables and Welch’s two-sample t test for continuous variables. CHARLS (China Health and Retirement Longitudinal Study); CNY, Chinese yuan; SD, standard deviation.

All 13 assessed non-diabetes LTCs were more prevalent in participants with diabetes than among those without diabetes (all *p* < 0.001). The most common conditions among participants with diabetes were hypertension (64.05%), dyslipidaemia (52.75%), and arthritis or rheumatism (45.00%). Participants with diabetes had a higher mean number of LTCs (4.18 vs. 1.88), a higher prevalence of multimorbidity (92.93% vs. 50.55%) and ≥3 LTCs (77.68% vs. 29.82%), and a higher mean CMWI score (5.80 vs. 2.90; all *p* < 0.001). The mean number of non-diabetes LTCs was also higher among participants with diabetes (3.18 vs. 1.88). Across all age groups, participants with diabetes had higher mean numbers of both total LTCs and non-diabetes LTCs (Figures 2A,B).

**Figure 2:**
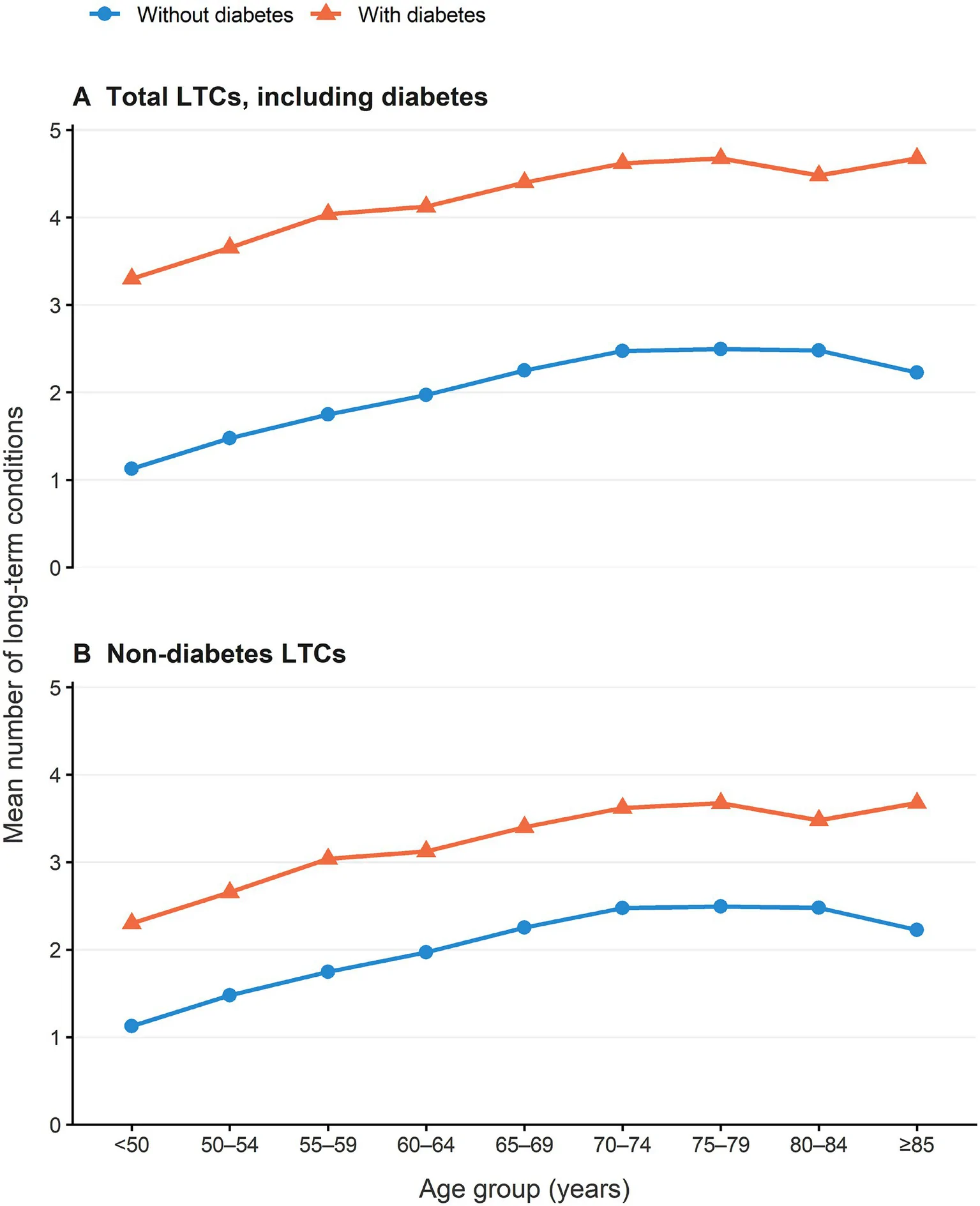
Mean number of long-term conditions by age group and diabetes status in CHARLS. **(A)** Total number of LTCs, including diabetes. **(B)** Number of non-diabetes LTCs, based on the same 13 non-diabetes condition categories in both groups. Points represent unweighted means within each age group. LTCs, long-term conditions.

Participants with diabetes also reported greater healthcare utilization and expenditure (Table 1). Inpatient utilization during the preceding year was reported by 27.14% of participants with diabetes and 15.32% of those without diabetes. Outpatient utilization during the preceding month was reported by 22.99 and 15.52%, respectively. Mean hospitalization counts were 0.47 versus 0.25, and mean outpatient visit counts were 0.51 versus 0.33 (both *p* < 0.001). Mean total and out-of-pocket expenditures for inpatient and outpatient services were higher among participants with diabetes (all *p* < 0.05).

The hospital cohort included 3,963 patients with T2DM, with a mean age of 56.06 ± 13.42 years; 42.59% were women (Supplementary Table S2). Recorded diabetes-related complications were present in 73.71%. Hypertension (38.78%) and kidney disease (38.43%) were the most prevalent coexisting conditions. The mean number of LTCs was 3.08 ± 1.48, and the mean CMWI score was 4.04 ± 3.11. Multimorbidity and ≥3 LTCs were present in 85.89 and 60.86% of patients, respectively.

### Associations with healthcare utilization in CHARLS

After covariate adjustment, all four multimorbidity measures were positively associated with inpatient and outpatient care utilization among CHARLS participants with diabetes (Table 2). Multimorbidity was associated with higher odds of inpatient utilization (OR, 2.22; 95% CI, 1.46–3.52) and outpatient utilization (OR, 2.95; 95% CI, 1.84–5.04). The corresponding ORs for ≥3 LTCs were 2.44 (95% CI, 1.90–3.16) and 2.17 (95% CI, 1.68–2.84). Higher LTC counts and CMWI scores were also associated with higher odds of both utilization outcomes.

**Table 2:** Associations of multimorbidity measures with healthcare utilization and expenditures among CHARLS participants with diabetes, overall and by sex.

Multimorbidity measure	Inpatient care utilization	Outpatient care utilization	Number of hospitalizations	Number of outpatient visits	Total inpatient expenditure	Out-of-pocket inpatient expenditure	Total outpatient expenditure	Out-of-pocket outpatient expenditure
	OR (95% CI)	OR (95% CI)	Count ratio (95% CI)	Count ratio (95% CI)	Cost ratio (95% CI)	Cost ratio (95% CI)	Cost ratio (95% CI)	Cost ratio (95% CI)
Overall								
Multimorbidity	2.22 (1.46–3.52)	2.95 (1.84–5.04)	2.87 (1.69–5.38)	2.75 (1.42–6.31)	2.31 (1.31–6.27)	2.81 (1.54–7.40)	5.16 (2.34–22.49)	5.46 (2.75–15.39)
≥3 long-term conditions	2.44 (1.90–3.16)	2.17 (1.68–2.84)	2.48 (1.87–3.37)	2.09 (1.48–3.04)	2.98 (1.94–5.24)	2.39 (1.27–5.52)	3.15 (1.92–5.72)	3.10 (1.92–5.06)
Number of long-term conditions	1.25 (1.19–1.30)	1.22 (1.17–1.28)	1.21 (1.16–1.25)	1.15 (1.09–1.21)	1.24 (1.15–1.32)	1.23 (1.14–1.33)	1.35 (1.22–1.47)	1.29 (1.18–1.39)
Chinese multimorbidity-weighted index	1.12 (1.09–1.14)	1.08 (1.06–1.11)	1.10 (1.08–1.12)	1.07 (1.04–1.09)	1.13 (1.09–1.17)	1.14 (1.09–1.18)	1.15 (1.09–1.20)	1.12 (1.07–1.17)
Male								
Multimorbidity	3.01 (1.65–6.07)	2.55 (1.39–5.15)	3.89 (1.77–11.07)	2.18 (0.89–7.35)	2.01 (0.68–16.33)	2.58 (0.76–18.71)	5.43 (2.42–34.25)	3.42 (1.53–18.56)
≥3 long-term conditions	2.57 (1.80–3.73)	2.56 (1.75–3.83)	2.77 (1.84–4.38)	2.70 (1.51–5.33)	3.01 (1.62–6.93)	3.63 (1.95–8.22)	3.80 (1.96–9.19)	3.01 (1.67–6.44)
Number of long-term conditions	1.28 (1.20–1.37)	1.23 (1.15–1.32)	1.21 (1.14–1.28)	1.09 (0.99–1.20)	1.27 (1.13–1.42)	1.32 (1.17–1.46)	1.31 (1.15–1.46)	1.21 (1.11–1.35)
Chinese multimorbidity-weighted index	1.14 (1.10–1.18)	1.10 (1.06–1.14)	1.11 (1.08–1.14)	1.05 (1.01–1.10)	1.14 (1.09–1.20)	1.17 (1.11–1.23)	1.14 (1.06–1.21)	1.11 (1.04–1.17)
Female								
Multimorbidity	1.61 (0.91–3.04)	3.63 (1.76–8.80)	2.14 (1.11–4.89)	3.80 (1.51–13.93)	1.81 (0.84–5.98)	1.88 (0.80–6.37)	4.51 (1.60–88.58)	5.31 (1.81–47.16)
≥3 long-term conditions	2.32 (1.64–3.35)	1.91 (1.36–2.76)	2.25 (1.55–3.42)	1.74 (1.18–2.68)	2.72 (1.50–5.90)	1.66 (0.76–5.28)	2.77 (1.44–6.93)	3.00 (1.61–6.35)
Number of long-term conditions	1.22 (1.15–1.30)	1.22 (1.15–1.30)	1.20 (1.14–1.26)	1.19 (1.13–1.27)	1.21 (1.13–1.31)	1.14 (1.04–1.27)	1.36 (1.21–1.52)	1.31 (1.18–1.48)
Chinese multimorbidity-weighted index	1.10 (1.07–1.14)	1.07 (1.04–1.10)	1.09 (1.06–1.12)	1.08 (1.04–1.11)	1.12 (1.07–1.16)	1.09 (1.04–1.15)	1.15 (1.08–1.22)	1.12 (1.06–1.20)
P for interaction by sex								
Multimorbidity × sex	0.124	0.621	0.240	0.549	0.326	0.250	0.318	0.658
≥3 long-term conditions × sex	0.601	0.190	0.400	0.180	0.864	0.092	0.381	0.500
Number of long-term conditions × sex	0.256	0.686	0.681	0.182	0.684	0.038	0.847	0.295
Chinese multimorbidity-weighted index × sex	0.126	0.129	0.195	0.642	0.498	0.017	0.286	0.188

Binary utilization outcomes were analyzed using logistic regression, and visit counts using quasi-Poisson regression with a log link. Expenditure outcomes were analyzed using two-part models combining logistic regression and Gamma regression with a log link; cost ratios represent ratios of standardized overall mean expenditure, including zero expenditure. Models were adjusted for age, education, residence, marital status, health insurance, and diabetes control, with additional adjustment for sex in overall analyses. Sex-stratified analyses and interaction tests were exploratory; interaction *p* values were unadjusted for multiple testing. CHARLS, China Health and Retirement Longitudinal Study; OR, odds ratio; CI, confidence interval.

All four measures were additionally associated with greater hospitalization and outpatient visit counts (Table 2). Multimorbidity was associated with count ratios of 2.87 (95% CI, 1.69–5.38) for hospitalizations and 2.75 (95% CI, 1.42–6.31) for outpatient visits. The corresponding ratios for ≥3 LTCs were 2.48 (95% CI, 1.87–3.37) and 2.09 (95% CI, 1.48–3.04). Each additional LTC was associated with 21% more hospitalizations and 15% more outpatient visits, while each 1-point increase in CMWI was associated with 10 and 7% higher counts, respectively.

### Associations with healthcare expenditures in CHARLS

All four multimorbidity measures were associated with higher overall mean inpatient and outpatient expenditures, including zero expenditure, after covariate adjustment (Table 2). Multimorbidity was associated with total and out-of-pocket inpatient cost ratios of 2.31 (95% CI, 1.31–6.27) and 2.81 (95% CI, 1.54–7.40), respectively. The corresponding outpatient cost ratios were 5.16 (95% CI, 2.34–22.49) and 5.46 (95% CI, 2.75–15.39). The presence of ≥3 LTCs was associated with total and out-of-pocket inpatient cost ratios of 2.98 (95% CI, 1.94–5.24) and 2.39 (95% CI, 1.27–5.52). Its corresponding outpatient cost ratios were 3.15 (95% CI, 1.92–5.72) and 3.10 (95% CI, 1.92–5.06). Each additional LTC was associated with 24% higher total inpatient expenditure, 23% higher out-of-pocket inpatient expenditure, 35% higher total outpatient expenditure, and 29% higher out-of-pocket outpatient expenditure. Each 1-point increase in CMWI was associated with 13%, 14%, 15%, and 12% higher expenditures, respectively.

Separate model components showed that all four measures were associated with higher odds of positive expenditure across the four outcomes (Supplementary Table S3). Among participants with positive expenditure, associations were less consistent: binary multimorbidity had no clear association with expenditure amounts, whereas CMWI was positively associated with all four expenditure outcomes. Positive-expenditure distributions and zero-expenditure proportions are shown in Supplementary Figure S1.

### Associations with inpatient outcomes in the hospital cohort

After covariate adjustment, all four multimorbidity measures were positively associated with hospitalization count in the hospital cohort (Table 3). Multimorbidity and ≥3 LTCs had mean parameter ratios of 4.19 (95% CI, 2.98–5.87) and 4.08 (95% CI, 3.33–5.01), respectively. Each additional LTC and each 1-point increase in CMWI had corresponding ratios of 1.67 (95% CI, 1.58–1.75) and 1.23 (95% CI, 1.20–1.27). These estimates refer to the underlying negative binomial mean parameter before truncation.

**Table 3:** Associations of multimorbidity measures with inpatient outcomes among hospitalized patients with T2DM, overall and by sex.

Multimorbidity measure	Hospitalization count	Cumulative inpatient days	Total inpatient expenditure
	Mean parameter ratio (95% CI)	Mean ratio (95% CI)	Cost ratio (95% CI)
Overall			
Multimorbidity	4.19 (2.98–5.87)	1.34 (1.25–1.43)	1.55 (1.44–1.67)
≥3 long-term conditions	4.08 (3.33–5.01)	1.39 (1.33–1.46)	1.58 (1.50–1.66)
Number of long-term conditions	1.67 (1.58–1.75)	1.17 (1.15–1.19)	1.22 (1.20–1.24)
Chinese multimorbidity-weighted index	1.23 (1.20–1.27)	1.07 (1.06–1.08)	1.09 (1.08–1.10)
Male			
Multimorbidity	3.75 (2.42–5.83)	1.33 (1.22–1.44)	1.49 (1.36–1.64)
≥3 long-term conditions	4.23 (3.22–5.56)	1.38 (1.30–1.47)	1.54 (1.44–1.64)
Number of long-term conditions	1.64 (1.53–1.76)	1.16 (1.14–1.18)	1.21 (1.18–1.23)
Chinese multimorbidity-weighted index	1.23 (1.18–1.27)	1.07 (1.06–1.08)	1.09 (1.07–1.10)
Female			
Multimorbidity	4.87 (2.86–8.27)	1.36 (1.22–1.51)	1.62 (1.44–1.83)
≥3 long-term conditions	3.90 (2.88–5.27)	1.40 (1.30–1.51)	1.64 (1.51–1.78)
Number of long-term conditions	1.69 (1.57–1.82)	1.19 (1.16–1.21)	1.24 (1.21–1.27)
Chinese multimorbidity-weighted index	1.23 (1.18–1.29)	1.07 (1.06–1.09)	1.09 (1.08–1.11)
P for interaction by sex			
Multimorbidity × sex	0.377	0.508	0.194
≥3 long-term conditions × sex	0.681	0.422	0.122
Number of long-term conditions × sex	0.408	0.045	0.040
Chinese multimorbidity-weighted index × sex	0.655	0.117	0.240

Hospitalization count was analyzed using zero-truncated negative binomial regression with a log link; ratios refer to the underlying negative binomial mean parameter before truncation. Cumulative inpatient days and total inpatient expenditure were analyzed using Gamma regression with a log link; ratios represent adjusted mean ratios. Overall models were adjusted for sex, age, insurance status, and diabetes complications; sex-stratified models omitted sex. Interaction rows report *p* values only. Sex-stratified analyses and interaction tests were exploratory; interaction *p* values were unadjusted for multiple testing. CI, confidence interval.

All four measures were also associated with greater mean cumulative inpatient days. Multimorbidity and ≥3 LTCs had mean ratios of 1.34 (95% CI, 1.25–1.43) and 1.39 (95% CI, 1.33–1.46), respectively. Each additional LTC was associated with 17% greater mean cumulative inpatient days, while each 1-point increase in CMWI was associated with 7% greater mean cumulative inpatient days.

Total inpatient expenditure was higher across all four measures. Multimorbidity and ≥3 LTCs had cost ratios of 1.55 (95% CI, 1.44–1.67) and 1.58 (95% CI, 1.50–1.66), respectively. Each additional LTC was associated with 22% higher mean expenditure (cost ratio, 1.22; 95% CI, 1.20–1.24), and each 1-point increase in CMWI with 9% higher mean expenditure (cost ratio, 1.09; 95% CI, 1.08–1.10).

### Sex-stratified associations and interaction tests

All sex-stratified analyses and interaction tests were exploratory. Sex-stratified estimates were generally positive in both cohorts (Tables 2, 3). In CHARLS, multimorbidity was associated with inpatient utilization among men (OR, 3.01; 95% CI, 1.65–6.07), whereas the estimate among women was less precise (OR, 1.61; 95% CI, 0.91–3.04). The corresponding interaction *p* value was 0.124. All interaction *p* values for binary utilization and visit counts were ≥0.05.

For CHARLS out-of-pocket inpatient expenditure, nominal interaction *p* values were below 0.05 for LTC count (P for interaction = 0.038) and CMWI (P for interaction = 0.017). The sex-specific overall cost ratios per additional LTC were 1.32 among men and 1.14 among women. Corresponding ratios per 1-point increase in CMWI were 1.17 and 1.09. All other expenditure interaction *p* values were ≥0.05.

In the hospital cohort, nominal interaction *p* values were below 0.05 for LTC count in relation to cumulative inpatient days (P for interaction = 0.045) and total inpatient expenditure (P for interaction = 0.040). Per additional LTC, the mean inpatient days ratios were 1.16 among men and 1.19 among women, and the cost ratios were 1.21 and 1.24, respectively. All remaining hospital interaction *p* values were ≥0.05. These interaction tests were unadjusted for multiple testing, and the nominal findings should be considered hypothesis-generating rather than confirmatory evidence of sex differences.

### Sensitivity analyses

In CHARLS, survey-weighted analyses retained positive associations between all four multimorbidity measures and healthcare utilization and expenditures (Supplementary Table S4). However, the association between ≥3 LTCs and out-of-pocket inpatient expenditure was imprecise (cost ratio, 1.80; 95% CI, 0.47–6.87). Additional adjustment for lifestyle variables produced associations in the same direction as the primary analyses. Excluding diabetes from the multimorbidity measures also retained positive associations across all eight outcomes, with all 95% CIs excluding 1.

In the hospital cohort, associations with hospitalization count, cumulative inpatient days, and total inpatient expenditure persisted after excluding diabetes from the multimorbidity measures (Supplementary Table S5). Additional adjustment for pandemic period modestly attenuated hospitalization count and expenditure estimates. For example, the mean parameter ratio for multimorbidity and hospitalization count changed from 4.19 to 3.87 (95% CI, 2.74–5.45), while the total inpatient cost ratio changed from 1.55 to 1.50 (95% CI, 1.39–1.62). All four multimorbidity measures remained positively associated with all three inpatient outcomes, with 95% CIs excluding 1.

## Discussion

This study examined associations between multimorbidity burden and healthcare utilization and expenditures among people with diabetes using CHARLS and a single-center hospital cohort. Among CHARLS participants with diabetes, multimorbidity and ≥3 LTCs were prevalent, affecting 92.93 and 77.68%, respectively. All four multimorbidity measures were associated with higher healthcare utilization and overall mean expenditures in the primary analyses. Positive associations were also observed for inpatient outcomes among hospitalized patients. These findings support attention to coexisting conditions when planning integrated care for people with diabetes (18, 19).

The higher burden of LTCs among participants with diabetes is consistent with the complex clinical profiles associated with the disease. Hyperglycemia, insulin resistance, and shared cardiometabolic risk factors may contribute to the coexistence of cardiovascular, renal, and neurological conditions (2, 20–22). We observed higher mean CMWI scores among participants with diabetes than among those without diabetes; however, diabetes itself was included in the multimorbidity measures and partly contributed to these differences. Importantly, associations with healthcare utilization and expenditures remained positive after excluding diabetes from the multimorbidity measures. Although previous longitudinal research has examined changes in multimorbidity burden over time (23, 24), our cross-sectional findings cannot establish temporal relationships or disease progression.

Greater multimorbidity burden was consistently associated with higher healthcare utilization. In CHARLS, each additional LTC was associated with 25% higher odds of inpatient utilization and 22% higher odds of outpatient utilization, together with greater numbers of hospitalizations and outpatient visits. Positive associations with hospitalization frequency and cumulative inpatient days were also observed in the hospital cohort. These findings are consistent with the greater healthcare needs that may accompany the management of multiple chronic conditions. Patients with multimorbidity may require more frequent monitoring, specialist consultations, and coordination of treatments across conditions (25, 26). Fragmented care may further complicate disease management and healthcare delivery (27). However, these potential mechanisms were not directly assessed in our study, and the hospital-based findings should be interpreted within the selected population of hospitalized patients.

Multimorbidity burden was also associated with higher medical expenditures, including out-of-pocket expenditures. In CHARLS, the presence of ≥3 LTCs was associated with higher overall mean total inpatient and outpatient expenditures, with cost ratios of 2.98 and 3.15, respectively. The two-part models provided further insight into these associations. All four multimorbidity measures were associated with higher odds of incurring positive medical expenditures, whereas associations with expenditure amounts among healthcare users were less consistent. Notably, binary multimorbidity showed no clear association with expenditure amounts among participants with positive expenditure, while CMWI was positively associated with all four expenditure outcomes. These findings indicate that greater multimorbidity burden was associated with a higher likelihood of incurring medical expenses and, for some measures, higher expenditure amounts among those who incurred costs. The financial implications of multimorbidity may extend beyond direct medical spending, as previous research has identified economic burden as a factor associated with medication safety engagement among older adults with cardiometabolic multimorbidity (28). Nevertheless, broader financial consequences, including catastrophic health expenditure and indirect costs, were not evaluated in our study.

Sex-stratified analyses generally showed positive associations between multimorbidity burden and healthcare outcomes in both men and women. In exploratory analyses, the point estimates for LTC count and CMWI in relation to out-of-pocket inpatient expenditure were larger among men in CHARLS, whereas those for LTC count in relation to cumulative inpatient days and total inpatient expenditure were slightly larger among women in the hospital cohort. Although the corresponding four interaction tests yielded nominal *p* values below 0.05, these *p* values were not adjusted for multiple testing. These observations therefore do not establish effect modification by sex and should be considered hypothesis-generating. Differences between the two study populations further limit direct comparisons of these patterns. Independent studies are needed to determine whether these associations differ by sex.

The use of multiple multimorbidity measures provides complementary perspectives on the burden of coexisting conditions (29). Binary multimorbidity and ≥3 LTCs identify patients meeting different disease-count thresholds, while LTC count and CMWI provide continuous measures of disease burden. Unlike simple disease counts, CMWI incorporates disease-specific weights derived from Chinese population data (14). The consistent associations observed across these measures suggest that different approaches to characterizing multimorbidity may be informative when assessing healthcare needs. However, the present study did not directly compare their predictive performance, and the findings do not establish the superiority of any particular measure. From a clinical and health policy perspective, systematic assessment of coexisting conditions may help inform integrated care and healthcare resource planning for people with diabetes (19, 30). Further research is needed to determine whether incorporating these measures into clinical assessment improves risk prediction or resource allocation.

The use of complementary survey and clinical data sources, outcome-specific regression models, and multiple sensitivity analyses strengthened this study. Associations generally remained positive after incorporating survey weights, expanding covariate adjustment, excluding diabetes from multimorbidity measures, and accounting for the pandemic period in the hospital cohort. Nevertheless, several limitations should be acknowledged. First, the observational design precludes causal inference. The CHARLS analyses were cross-sectional, and temporal relationships between LTCs and healthcare outcomes could not be fully established. Greater healthcare utilization may also increase opportunities for detecting coexisting conditions. Second, CHARLS relied partly on self-reported diagnoses and healthcare utilization, which may be affected by recall bias and misclassification (31). Diabetes types could not be reliably distinguished in CHARLS; therefore, the survey findings should be interpreted as relating to diabetes overall rather than specifically to T2DM. The primary CHARLS analyses were unweighted, although survey-weighted sensitivity analyses were conducted, and the findings should not be interpreted as design-based national estimates. Third, the hospital cohort included only previously hospitalized patients from a single center, potentially introducing selection or collider bias and limiting generalizability. The zero-truncated model accounted for the absence of zero hospitalization counts but could not eliminate selection bias. Fourth, although the models adjusted for available covariates, diabetes severity was incompletely characterized, and residual confounding by disease duration, treatment, and other unmeasured factors remains possible. Finally, the differences in outcome definitions and observation periods between the two data sources limit direct quantitative comparisons. These limitations warrant cautious interpretation and further investigation using longitudinal and more broadly representative data.

## Conclusion

Among Chinese adults with diabetes, greater multimorbidity burden was associated with higher healthcare utilization and medical expenditures in both survey and hospital-based analyses. Different multimorbidity measures provided complementary information on these associations, highlighting the importance of considering coexisting conditions in healthcare planning. Further longitudinal research is needed to clarify temporal relationships and evaluate the clinical utility of these measures.

## Data Availability

CHARLS data can be accessed from the website (https://charls.pku.edu.cn/). The hospital cohort data from Chinese patients with diabetes are available from the corresponding author upon reasonable request.
