# Supplementary material for: Associations of multimorbidity burden with healthcare utilization and expenditures among individuals with diabetes in China
Source: Front Public Health. 2026 Oct 28;14:1975609. doi: 10.3389/fpubh.2026.1975609 (PMC13751521; doi:10.3389/fpubh.2026.1975609)
Supplement: Supplementary file 1 [file Supplementary_file_1.DOCX]

**Figure S1. Distributions of healthcare utilization and expenditure outcomes among CHARLS participants and hospitalized patients with T2DM.**

Panels A–F show outcomes among CHARLS participants with diabetes (n = 2,531): hospitalization count (A), outpatient visit count (B), total inpatient expenditure (C), out-of-pocket inpatient expenditure (D), total outpatient expenditure (E), and out-of-pocket outpatient expenditure (F). Panels G–I show hospitalization count (G), cumulative inpatient days (H), and total inpatient expenditure (I) among hospitalized patients with T2DM (n = 3,963). CHARLS inpatient outcomes refer to the past year, and outpatient outcomes to the past month; hospital cohort outcomes include the index hospitalization and subsequent hospitalizations during the one-year follow-up after the first discharge. Expenditure histograms include only participants with positive expenditure and use logarithmic x-axes; zero-expenditure counts and percentages are indicated within each panel. Percentages are unweighted and calculated within the sample displayed in each panel. CNY, Chinese yuan; T2DM, type 2 diabetes mellitus.

**Table S1. Conditions and corresponding ICD-10 codes used in the hospital cohort**

| **Condition** | **Conditions included** | **ICD-10 codes** |
| --- | --- | --- |
| Diabetes | Type 1, type 2, and other or unspecified diabetes | E10–E14 |
| Hypertension | Essential, secondary, and hypertensive heart/renal diseases; excluding pulmonary and pregnancy-related hypertension | I10–I15 |
| Dyslipidaemia | Disorders of lipoprotein metabolism and other lipidaemias | E78 |
| Cancer or malignant tumor | Malignant neoplasms | C00–C97 |
| Chronic lung diseases | Chronic bronchitis, emphysema, COPD, and bronchiectasis | J40–J44, J47 |
| Liver disease | Chronic viral hepatitis and alcoholic, toxic, inflammatory, or other liver diseases | K70–K77, B18 |
| Heart disease | Ischaemic heart disease, heart failure, and other cardiac disorders | I20–I25, I30–I52 |
| Stroke | Cerebral haemorrhage, infarction, other cerebrovascular diseases, and their sequelae | I60–I69 |
| Kidney disease | Glomerular and tubulointerstitial diseases, renal failure, urinary stones, and other kidney/ureter disorders | N00–N29 |
| Stomach or other digestive disease | Diseases of the oesophagus, stomach, and duodenum | K20–K31 |
| Emotional, nervous, or psychiatric problems | Mental and behavioural disorders, excluding dementia coded F00–F03 | F04–F99 |
| Memory-related diseases | Alzheimer’s disease and dementia | F00–F03, G30 |
| Arthritis or rheumatism | Rheumatoid arthritis and osteoarthritis | M05–M06, M15–M19 |
| Asthma | Asthma and status asthmaticus | J45–J46 |

**Table S2. Characteristics of 3,963 hospitalized patients with T2DM**

| **Characteristic** | **N = 3,963***^1^* |
| --- | --- |
| Sex (female) | 1,688 (42.59%) |
| Age (years) | 56.06 ± 13.42 |
| Public health insurance | 3,796 (95.79%) |
| Diabetes-related complications | 2,921 (73.71%) |
| Hypertension | 1,537 (38.78%) |
| Dyslipidaemia | 1,208 (30.48%) |
| Cancer or malignant tumor | 451 (11.38%) |
| Chronic lung diseases | 778 (19.63%) |
| Liver disease | 351 (8.86%) |
| Heart disease | 525 (13.25%) |
| Stroke | 378 (9.54%) |
| Kidney disease | 1,523 (38.43%) |
| Stomach or other digestive disease | 850 (21.45%) |
| Emotional, nervous, or psychiatric problems | 62 (1.56%) |
| Memory-related diseases | 230 (5.80%) |
| Arthritis or rheumatism | 303 (7.65%) |
| Asthma | 32 (0.81%) |
| Number of long-term conditions | 3.08 ± 1.48 |
| Multimorbidity | 3,404 (85.89%) |
| ≥3 long-term conditions | 2,412 (60.86%) |
| Chinese multimorbidity-weighted index | 4.04 ± 3.11 |
| Hospitalization frequency | 1.31 ± 0.77 |
| Cumulative inpatient days | 11.59 ± 9.23 |
| Total inpatient expenditure (CNY) | 14,073.16 ± 12,366.36 |
| Year of hospitalization |  |
| 2018 | 415 (10.47%) |
| 2019 | 471 (11.88%) |
| 2020 | 690 (17.41%) |
| 2021 | 736 (18.57%) |
| 2022 | 1,309 (33.03%) |
| 2023 | 342 (8.63%) |
| Data are presented as n (%) or mean ± SD. SD, standard deviation; CNY, Chinese yuan; T2DM, type 2 diabetes mellitus. | |
|  |  |

| **Table S3. Two-part model results for healthcare expenditures among CHARLS participants with diabetes** | | | | |
| --- | --- | --- | --- | --- |
| **Multimorbidity measure** | **Part 1: OR (95% CI)** | ***P* value** | **Part 2: Cost ratio (95% CI)** | ***P* value** |
| **Total inpatient expenditure — Part 1: n = 2,531; Part 2: n = 643** |  |  |  |  |
| Multimorbidity | 2.06 (1.33–3.18) | 0.001 | 1.31 (0.62–2.78) | 0.484 |
| ≥3 long-term conditions | 2.29 (1.77–2.96) | <0.001 | 1.56 (1.01–2.42) | 0.044 |
| Number of long-term conditions | 1.24 (1.19–1.30) | <0.001 | 1.07 (1.00–1.15) | 0.052 |
| Chinese multimorbidity-weighted index | 1.11 (1.09–1.14) | <0.001 | 1.05 (1.01–1.09) | 0.007 |
| **Out-of-pocket inpatient expenditure — Part 1: n = 2,531; Part 2: n = 639** |  |  |  |  |
| Multimorbidity | 2.05 (1.32–3.18) | 0.001 | 1.59 (0.68–3.71) | 0.279 |
| ≥3 long-term conditions | 2.31 (1.79–2.99) | <0.001 | 1.24 (0.75–2.06) | 0.402 |
| Number of long-term conditions | 1.24 (1.18–1.30) | <0.001 | 1.07 (0.99–1.15) | 0.111 |
| Chinese multimorbidity-weighted index | 1.12 (1.09–1.14) | <0.001 | 1.06 (1.02–1.10) | 0.004 |
| **Total outpatient expenditure — Part 1: n = 2,531; Part 2: n = 543** |  |  |  |  |
| Multimorbidity | 2.88 (1.73–4.82) | <0.001 | 2.10 (0.69–6.35) | 0.188 |
| ≥3 long-term conditions | 2.14 (1.63–2.80) | <0.001 | 1.69 (0.98–2.92) | 0.061 |
| Number of long-term conditions | 1.21 (1.15–1.27) | <0.001 | 1.18 (1.09–1.28) | 0.001 |
| Chinese multimorbidity-weighted index | 1.08 (1.05–1.10) | <0.001 | 1.09 (1.05–1.14) | <0.001 |
| **Out-of-pocket outpatient expenditure — Part 1: n = 2,531; Part 2: n = 527** |  |  |  |  |
| Multimorbidity | 2.80 (1.68–4.69) | <0.001 | 2.27 (0.86–6.04) | 0.099 |
| ≥3 long-term conditions | 2.14 (1.63–2.81) | <0.001 | 1.66 (1.02–2.71) | 0.043 |
| Number of long-term conditions | 1.21 (1.16–1.27) | <0.001 | 1.12 (1.04–1.21) | 0.005 |
| Chinese multimorbidity-weighted index | 1.08 (1.05–1.10) | <0.001 | 1.06 (1.02–1.11) | 0.006 |
| Part 1 used logistic regression to model the probability of positive expenditure. Part 2 used Gamma regression with a log link among participants with positive expenditure; cost ratios represent adjusted mean expenditure ratios in this subgroup. Models were adjusted for sex, age, education, residence, marital status, health insurance, and diabetes control. Each multimorbidity measure was fitted separately. CI, confidence interval; OR, odds ratio. | | | | |
|  |  |  |  |  |
|  |  |  |  |  |

| **Table S4. Sensitivity analyses of associations between multimorbidity measures and healthcare utilization and expenditures among CHARLS participants with diabetes** | | | | | | | | |
| --- | --- | --- | --- | --- | --- | --- | --- | --- |
| **Multimorbidity measure** | **Inpatient care utilization** | **Outpatient care utilization** | **Number of hospitalizations** | **Number of outpatient visits** | **Total inpatient expenditure** | **Out-of-pocket inpatient expenditure** | **Total outpatient expenditure** | **Out-of-pocket outpatient expenditure** |
|  | OR (95% CI) | OR (95% CI) | Count ratio (95% CI) | Count ratio (95% CI) | Cost ratio (95% CI) | Cost ratio (95% CI) | Cost ratio (95% CI) | Cost ratio (95% CI) |
| **A. Survey-weighted analyses** |  |  |  |  |  |  |  |  |
| Multimorbidity | 2.81 (1.78–4.43) | 2.66 (1.47–4.85) | 3.22 (2.04–5.07) | 2.48 (1.38–4.46) | 3.82 (1.90–7.68) | 3.94 (1.58–9.82) | 4.60 (1.41–15.03) | 4.51 (1.87–10.88) |
| ≥3 long-term conditions | 2.73 (1.96–3.81) | 2.95 (2.06–4.22) | 2.66 (1.84–3.83) | 2.55 (1.81–3.59) | 4.15 (2.68–6.42) | 1.80 (0.47–6.87) | 5.23 (2.96–9.24) | 4.10 (2.32–7.24) |
| Number of long-term conditions | 1.28 (1.21–1.36) | 1.25 (1.19–1.32) | 1.22 (1.18–1.28) | 1.17 (1.10–1.24) | 1.28 (1.19–1.38) | 1.22 (1.07–1.39) | 1.47 (1.31–1.66) | 1.34 (1.21–1.48) |
| Chinese multimorbidity-weighted index | 1.13 (1.10–1.17) | 1.09 (1.06–1.12) | 1.10 (1.08–1.13) | 1.07 (1.04–1.10) | 1.14 (1.10–1.18) | 1.14 (1.07–1.21) | 1.21 (1.13–1.29) | 1.16 (1.09–1.23) |
| **B. Additional adjustment for lifestyle variables** |  |  |  |  |  |  |  |  |
| Multimorbidity | 2.14 (1.38–3.33) | 2.93 (1.78–4.83) | 2.72 (1.57–4.72) | 2.69 (1.29–5.61) | 1.98 (1.08–5.11) | 2.16 (1.12–5.60) | 4.70 (2.01–20.07) | 4.91 (2.27–13.29) |
| ≥3 long-term conditions | 2.39 (1.85–3.08) | 2.16 (1.66–2.81) | 2.39 (1.81–3.18) | 2.04 (1.43–2.93) | 3.01 (2.04–5.10) | 2.44 (1.45–4.73) | 3.07 (1.91–5.18) | 2.87 (1.83–4.80) |
| Number of long-term conditions | 1.24 (1.18–1.29) | 1.23 (1.17–1.29) | 1.19 (1.15–1.23) | 1.14 (1.08–1.21) | 1.22 (1.15–1.30) | 1.20 (1.12–1.30) | 1.33 (1.20–1.45) | 1.26 (1.15–1.36) |
| Chinese multimorbidity-weighted index | 1.11 (1.09–1.14) | 1.08 (1.06–1.11) | 1.09 (1.07–1.11) | 1.06 (1.04–1.09) | 1.11 (1.08–1.15) | 1.12 (1.08–1.16) | 1.15 (1.08–1.20) | 1.11 (1.06–1.16) |
| **C. Exclusion of diabetes from multimorbidity measures** |  |  |  |  |  |  |  |  |
| Multimorbidity | 2.44 (1.89–3.14) | 2.17 (1.67–2.83) | 2.48 (1.85–3.33) | 2.09 (1.46–2.99) | 2.98 (1.93–5.04) | 2.39 (1.31–5.50) | 3.15 (1.92–5.71) | 3.10 (2.00–5.12) |
| ≥3 non-diabetes long-term conditions | 2.48 (2.04–3.02) | 1.83 (1.50–2.24) | 2.38 (1.91–2.96) | 1.52 (1.17–1.97) | 2.72 (1.92–4.07) | 2.64 (1.66–4.30) | 3.19 (2.22–4.66) | 2.91 (2.01–4.17) |
| Number of long-term conditions | 1.25 (1.19–1.30) | 1.22 (1.17–1.28) | 1.21 (1.16–1.25) | 1.15 (1.09–1.21) | 1.24 (1.16–1.32) | 1.23 (1.14–1.32) | 1.35 (1.23–1.47) | 1.29 (1.19–1.39) |
| Chinese multimorbidity-weighted index | 1.12 (1.09–1.14) | 1.08 (1.06–1.11) | 1.10 (1.08–1.12) | 1.07 (1.04–1.10) | 1.13 (1.09–1.16) | 1.14 (1.10–1.18) | 1.15 (1.09–1.21) | 1.12 (1.07–1.17) |
| Binary utilization outcomes were analyzed using logistic regression, and counts using quasi-Poisson regression with a log link. Expenditure outcomes were analyzed using two-part models combining logistic regression and Gamma regression with a log link; cost ratios represent standardized overall mean expenditure ratios, including zero expenditure. The primary adjustment set included sex, age, education, residence, marital status, health insurance, and diabetes control. A: Analyses incorporated 2018 individual survey weights, community clustering, and county-level stratification as an approximate survey-design specification. Cost confidence intervals were calculated using bootstrap replicate-weight standard errors on the log-ratio scale. B: Unweighted models additionally adjusted for smoking, drinking, social activity, physical activity, and sleep duration. C: Diabetes was excluded from all multimorbidity measures. Multimorbidity and ≥3 long-term conditions were defined using thresholds of at least two and at least three non-diabetes long-term conditions, respectively. A weight of 1 for diabetes was subtracted from CMWI. Models used the primary adjustment set. For B and C, cost confidence intervals were calculated using 1,000 individual-level bootstrap resamples. CI, confidence interval; OR, odds ratio. | | | | | | | | |
|  |  |  |  |  |  |  |  |  |
|  |  |  |  |  |  |  |  |  |
|  |  |  |  |  |  |  |  |  |
|  |  |  |  |  |  |  |  |  |
|  |  |  |  |  |  |  |  |  |

| **Table S5. Sensitivity analyses of associations between multimorbidity measures and inpatient outcomes among hospitalized patients with T2DM** | | | |
| --- | --- | --- | --- |
| **Multimorbidity measure** | **Hospitalization count** | **Cumulative inpatient days** | **Total inpatient expenditure** |
|  | Mean parameter ratio (95% CI) | Mean ratio (95% CI) | Cost ratio (95% CI) |
| **A. Exclusion of diabetes from multimorbidity measures** |  |  |  |
| Multimorbidity | 4.08 (3.33–5.01) | 1.39 (1.33–1.46) | 1.58 (1.50–1.66) |
| ≥3 non-diabetes long-term conditions | 4.08 (3.48–4.80) | 1.49 (1.42–1.56) | 1.65 (1.56–1.73) |
| Number of long-term conditions | 1.67 (1.58–1.75) | 1.17 (1.15–1.19) | 1.22 (1.20–1.24) |
| Chinese multimorbidity-weighted index | 1.23 (1.20–1.27) | 1.07 (1.06–1.08) | 1.09 (1.08–1.10) |
| **B. Additional adjustment for pandemic period** |  |  |  |
| Multimorbidity | 3.87 (2.74–5.45) | 1.34 (1.25–1.43) | 1.50 (1.39–1.62) |
| ≥3 long-term conditions | 3.63 (2.97–4.43) | 1.39 (1.33–1.46) | 1.54 (1.46–1.62) |
| Number of long-term conditions | 1.60 (1.52–1.68) | 1.17 (1.16–1.19) | 1.21 (1.19–1.23) |
| Chinese multimorbidity-weighted index | 1.21 (1.17–1.24) | 1.07 (1.06–1.08) | 1.09 (1.08–1.10) |
| Hospitalization count was analyzed using zero-truncated negative binomial regression with a log link; reported ratios refer to the underlying negative binomial mean parameter before truncation. Cumulative inpatient days and total inpatient expenditure were analyzed using Gamma regression with a log link; reported ratios represent adjusted mean ratios. All models were adjusted for sex, age, insurance status, and recorded diabetes-related complications. Each multimorbidity measure was fitted separately. A: Diabetes was excluded from all multimorbidity measures. Multimorbidity and ≥3 long-term conditions were defined using thresholds of at least two and at least three non-diabetes long-term conditions, respectively. A weight of 1 for diabetes was subtracted from CMWI. B: Models additionally adjusted for pandemic period, categorized as 2018–2019 versus 2020–2023. CI, confidence interval. | | | |
|  |  |  |  |
|  |  |  |  |
|  |  |  |  |
|  |  |  |  |
|  |  |  |  |
